# A multi-targeting natural compound with growth inhibitory and anti-angiogenic properties re-sensitizes chemotherapy resistant cancer

**DOI:** 10.1371/journal.pone.0218125

**Published:** 2019-06-11

**Authors:** Wesley F. Taylor, Sara E. Moghadam, Mahdi Moridi Farimani, Samad N. Ebrahimi, Marzieh Tabefam, Ehsan Jabbarzadeh

**Affiliations:** 1 Department of Chemical Engineering, University of South Carolina, Columbia, South Carolina, United States of America; 2 Department of Phytochemistry, Medicinal Plants and Drugs Research Institute, Shahid Beheshti University, G. C., Evin, Tehran, Iran; 3 Biomedical Engineering Program, University of South Carolina, Columbia, South Carolina, United States of America; University of South Alabama Mitchell Cancer Institute, UNITED STATES

## Abstract

Targeted therapies have become the focus of much of the cancer therapy research conducted in the United States. While these therapies have made vast improvements in the treatment of cancer, their results have been somewhat disappointing due to acquired resistances, high cost, and limited populations of susceptible patients. As a result, targeted therapeutics are often combined with other targeted therapeutics or chemotherapies. Compounds which target more than one cancer related pathway are rare, but have the potential to synergize multiple components of therapeutic cocktails. Natural products, as opposed to targeted therapies, typically interact with multiple cellular targets simultaneously, making them a potential source of synergistic cancer treatments. In this study, a rare natural product, deacetylnemorone, was shown to inhibit cell growth in a broad spectrum of cancer cell lines, selectively induce cell death in melanoma cells, and inhibit angiogenesis and invasion. Combined, these results demonstrate that deacetylnemorone affects multiple cancer-related targets associated with tumor growth, drug resistance, and metastasis. Thus, the multi-targeting natural product, deacetylnemorone, has the potential to enhance the efficacy of current cancer treatments as well as reduce commonly acquired treatment resistance.

## Introduction

Cancer remains the second leading cause of death in the United States according to the Centers for Disease Control and Prevention[1]. In recent years, there has been a shift in research efforts focusing on cancer drug discovery from cytotoxic chemotherapy agents, which induce cell death in rapidly dividing cells relatively indiscriminately, to targeted therapeutics, which influence specific cancer-related pathways. Targeted therapies have changed the landscape of cancer treatment from immune modulating therapies (such as monoclonal antibodies[2], cytokines[3], dendritic cell therapies[4], chimeric antigen receptor T cells (CAR-T cells)[5], and immune checkpoint blockade therapies[6]) to kinase inhibitors (including cyclin dependent kinase inhibitors[7], tyrosine kinase inhibitors[8], and phosphoinositide 3-kinase (PI3K) inhibitors[9]). Targeted therapies such as bevacizumab, sorafenib, ziv-aflibercept, and vandetanib have also emerged to inhibit angiogenesis, a process of new blood vessel formation, that is sometimes hijacked by cancer to feed growing and newly formed tumors[10, 11].

While these targeted therapies have led to a surge of improved prognoses, they have also come with drawbacks limiting their success in treating patients. For example, immune modulating targeted therapies, including sipuleucel-T and tisagenlecleucel, which activate the immune system against cancer by isolating immune cells from the patient’s body, altering their activity, and re-introducing the cells back into the patient[12, 13], can cost hundreds of thousands of dollars per injection[13], and come with strong side effects, including neurotoxicity, high fever, and respiratory distress[14]. Other targeted therapies, such as the anti-programmed cell death protein 1 (PD-1) drug nivolumab are less patient-tailored but suffer from a high risk of developed resistance and a low population of susceptible patients[15]. Similarly, therapies targeting cancer cell growth, such as tyrosine kinase inhibitors, often suffer from acquired resistance following the first few rounds of treatment. Angiogenesis targeting therapies trigger treatment resistance as a result of plasticity of the tumor microenvironment [10], upregulation of pro-angiogenic factors[16], recruitment of pro-angiogenic cells[17], and increased pericyte coverage[18]. Angiogenesis-targeting therapies also lead to increased hypoxia in the tumor microenvironment, resulting in increased tumor aggression and resistance to radiotherapy and chemotherapy [19, 20].

As a result of these challenges, targeted therapies are often administered in combination or in conjunction with chemotherapies in order to limit resistance and increase efficacy. The shortcomings of targeted therapies have led to a renewed interest in natural products for cancer treatment[21]. Compounds like natural products which are capable of targeting multiple cancer associated pathways may provide a more robust cancer treatment by limiting treatment acquired resistance, increasing the efficacy of multiple components of cancer therapy cocktails, and reducing the amount of drugs that are necessary to administer in order to achieve a positive treatment response.

In addition to influencing multiple biological targets, natural products are typically low cost and are associated with limited side effects. They have played an historically important role in cancer treatment, making up or inspiring approximately 60% of cancer treatments between 1981 and 2006[22, 23]. A select set of natural products have been identified that affect multiple cancer-related pathways simultaneously. For example, curcumin[24], emodin[25, 26], and astragaloside IV[27, 28], are thought to be capable of inducing apoptosis in cancer cells in addition to modulating the immune response to tumor formation. Curcumin[29] has also been suggested, along with resveratrol[30] and green tea catechins[31], as compounds that combine anti-proliferative and anti-angiogenic effects[32]. The widely used natural product derived chemotherapeutic, taxol, which targets microtubules, may owe some of its success to an ability to inhibit angiogenesis through vascular endothelial cell growth factor (VEGF) suppression at low concentrations[33]. By affecting multiple cancer related pathways simultaneously, natural products may be able to synergize with multiple components of commonly used mixtures of targeted therapies and chemotherapeutics, enhancing their efficacy and limiting resistance[34]. However, of the natural products that exhibit multi-targeted effects against cancer, many suffer from low bioavailability and low efficacy at low concentrations, necessitating the continued search for lead compounds.

Deacetylnemorone is a natural product of the abietane diterpenoid family that has been isolated from plants belonging to the genus *Salvia*. Like others from this class of natural compounds, deacetylnemorone has been shown to exhibit growth inhibitory properties in cancer cells, namely cervical and prostate cancer[35]. However, the anti-proliferative effect of this compound has not been widely established, nor has any mechanism of action been suggested. Furthermore, the effect of deacetylnemorone on other cancer related pathways has not been fully explored. Herein, the anti-proliferative effect of deacetylnemorone on multiple cancer tissue types, in addition to the effect of deacetylnemorone on angiogenesis and cancer cell invasion is studied. Cell cycle analysis was performed to gain insight into the compound’s mechanism of action. Finally, the ability of deacetylnemorone to enhance the cytotoxic effect of chemotherapy in treatment-resistant cancer cells was determined.

## Materials and methods

### Deacetylnemorone source and identification

**Fig 1:**
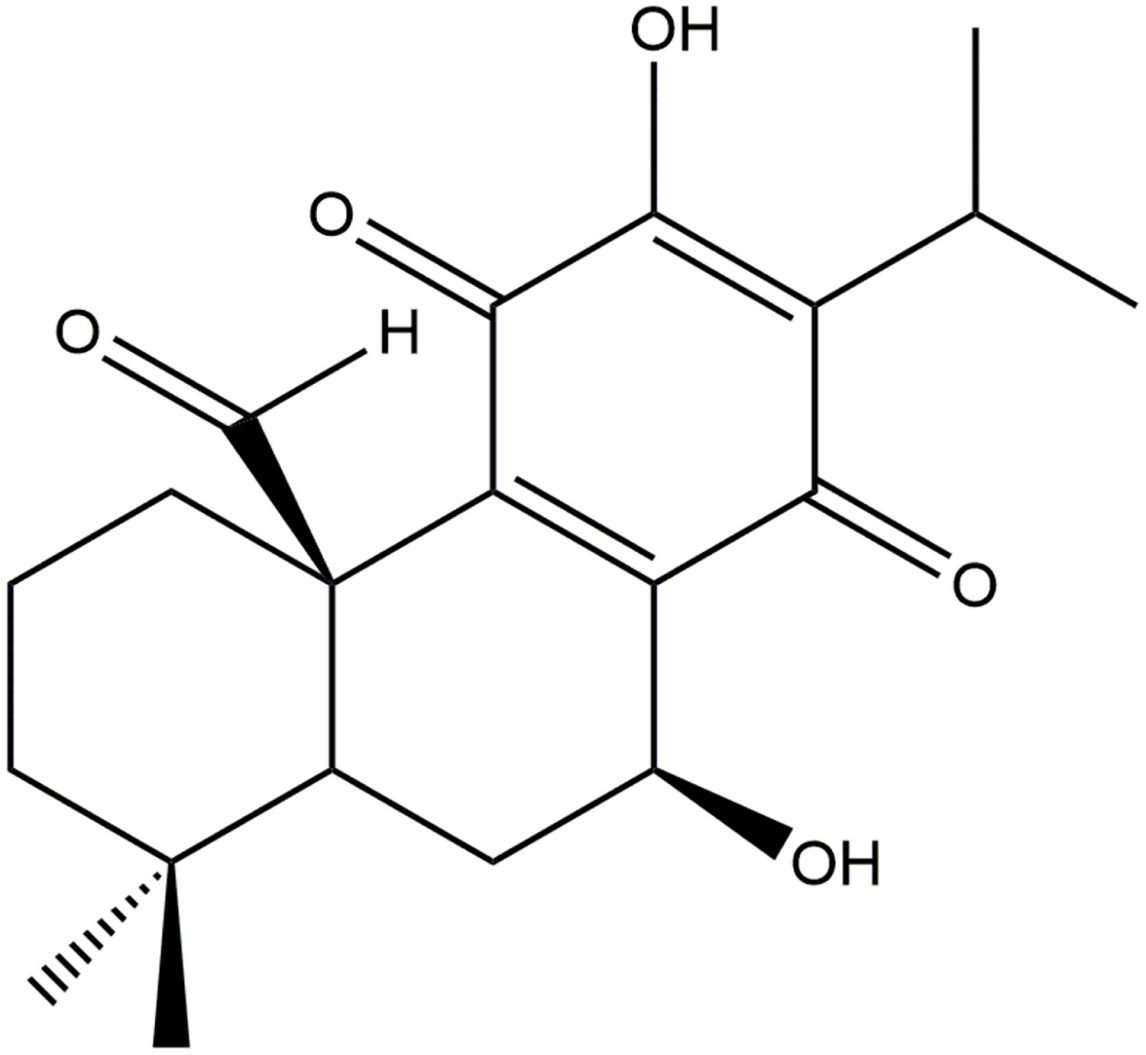
The structure of the abietane diterpenoid, deacetylnemorone.

Dried roots of *Salvia lerifolia* was extracted with hexane at room temperature followed by filtration and evaporation to afford dark brown extract. The dried extract was subjected to silica gel open column chromatography and washed with a gradient of non-polar to polar solvents. Deacetylnemorone was isolated and purified as a crystal[36]. The structure of the compound, shown in Fig 1, was determined by 1D and 2D nuclear magnetic resonance (NMR) in addition to time of flight mass spectrometry (TOF-MS). NMR analysis of deacetylnemorone was conducted using dimethyl sulfoxide (DMSO) as the solvent and a Bruker Avance III-HD 400 MHz. ^1^H-NMR, ^13^C-NMR, H-H correlation spectroscopy (COSY), heteronuclear single quantum coherence (HSQC), and heteronuclear multiple bond correlation (HMBC) were performed for structural determinations. For mass spectrophotometry analysis, deacetylnemorone was dissolved in methanol and analyzed using liquid chromatography-mass spectrometry on a Thermo Orbitrap Velos Pro. The generated spectra can be seen in S1A–S1E and S2A and S2B Figs. NMR assignment was provided in S1 Table. A stock solution of deacetylnemorone in DMSO at a concentration of 20mM was used for all cell culture experiments.

### NCI-60 screening

Primary cytotoxicity screening of deacetylnemorone against 59 cancer cell lines was performed using the National Institutes of Health’s (NIH) National Cancer Institute-60 (NCI-60) screening program[37]. This assay utilizes a Sulforhodamine B viability assay described by Shoemaker[38] to assess the growth percent of 60 immortalized cancer cell lines across 9 tissue types. One dose, 10 μM, of deacetylnemorone was tested. Only data generated from 59 cell lines was reported, as the HOP-92 non-small cell lung cancer cell line was excluded from the one dose screen. Growth percent between 0% and 100% in response to a compound can be interpreted as growth inhibition, and a negative percent growth is interpreted as cell death.

### Cell culture

MG-63 (osteosarcoma), SK-OV-3 (ovarian adenocarcinoma), MDA-MB-231 (breast cancer), HCT 116 (colorectal carcinoma), HCT 116/200 (FdUrd resistant subclone of HCT 116 cells), A2780ADR (doxorubicin resistant subclone of the ovarian carcinoma A2780), and HUVEC (normal human umbilical vein endothelial cells) were obtained and stored in liquid nitrogen until use. MG-63, SK-OV-3, MDA-MB-231, and HCT 116 cell lines were purchased from ATCC. A2780ADR cells were purchased from Sigma-Aldrich. HCT 116/200 cells were generously provided by Dr. Franklin G. Berger from the Center for Colon Cancer Research, where they were originally cultured[39]. Human umbilical vein cells (HUVEC) were purchased from Lonza. The culture media used for MG-63 was minimum essential medium (MEM;Corning) supplemented with 10% Fetal Bovine Essence (FBE;VWR) and 1% penicillin/streptomycin solution (Corning). The culture media for SK-OV-3 cells was McCoy’s 5A Medium (Sigma) supplemented with 10% FBE and 1% penicillin/streptomycin. The growth media for A2780ADR cells was Roswell Park Memorial Institute (RPMI) 1640 medium (Corning) supplemented with 10% FBE and 2mM L-glutamine (Thermo Fisher). The growth media used for MDA-MB-231, HCT 116, and HCT 116/200 cells was RPMI 1640 medium supplemented with 10% FBE and 1% penicillin/streptomycin. The growth media for HUVEC cells was endothelial cell growth medium-2 (EGM-2; Lonza Bullet Kit). All cells were maintained at 37 °C and 5% CO_2_.

### MTS assay

Cells were grown to approximately 80% confluency before being washed with phosphate buffered saline (PBS; Corning) and trypsinized using a 0.25% trypsin, 2.21 mM Ethylenediaminetetraacetic acid (EDTA), and sodium bicarbonate solution (Corning). Trypsinized cells were suspended in culture media and centrifuged at 2500 rpm for 5 minutes. Cell viability was confirmed using trypan blue (Gibco). Cells were then seeded into 96 well plates (VWR) with four replicates for each treatment group. MG-63, SK-OV-3, and A2780ADR cells were seeded at a density of 2,000 cells/well. MDA-MB-231 cells were seeded at a density of 5,000 cells/well. HCT 116 and HCT 116/200 cells were seeded at a density of 4,000 cells/well. HUVEC cells were seeded at a density of 3,000 cells/well. For all cell types, 100 μL of cell culture media was used. The cells were then incubated for 24 hours at 37 °C and 5% CO_2_ to allow for cell attachment. After cell attachment, the culture media was aspirated and replaced with media containing deacetylnemorone or DMSO. The vehicle control was 0.5% DMSO (Macron Fine Chemicals) in culture media. Doxorubicin hydrochloride (DOX;Sigma), and 5-fluoro-2’-deoxyuridine (FdUrd; Sigma), were used as positive controls. In combination studies, deacetylnemorone and FdUrd were added to the same culture media then added to the cells. At 48 and 72 hours, the cells were washed with PBS and culture media supplemented with 20% 3-(4,5-dimethylthiazol-2-yl)-5-(3-carboxymethoxyphenyl)-2-(4-sulfophenyl)-2H-tetrazolium (MTS) solution (Promega) was added to the cells. The cells were incubated for 2 hours, and the absorbance of each well at 490 nm was measured using a Spectramax 190 microplate reader.

### Cell cycle analysis

The effect of deacetylnemorone on the cell cycle of SK-MEL-5 melanoma cells was determined using flow cytometry. First, cells were seeded into 6 well plates at a density of 250,000 cells per well suspended in 2 mL of media. The cells were allowed to attach overnight, then the media was replaced with media containing deacetylnemorone. The cells were trypsinized with 0.25% trypsin and collected along with the drugged media at 6, 12, 24, 48, and 72 hours of treatment with deacetylnemorone. The detached cells were centrifuged at 2500 rpm for 5 minutes and washed with ice cold PBS twice. After centrifuging and discarding the supernatant, the pellet was suspended in 1 mL of ice-cold PBS, which was then added dropwise to 3 mL of ice-cold 70% ethanol in deionized water. The cells were fixed in this condition at 4° for at least 24 hours. After fixation, the cells were centrifuged, the supernatant was discarded, and the pellet was suspended in FxCycle PI/RNase Staining Solution (Invitrogen) for 15 minutes. The final cell suspension was analyzed using a BD LSR II flow cytometer. The percentage of cells in the sub-G1, G0/G1, S, and G2/M phases of the cell cycle were determined using the resulting histograms.

### In vitro invasion assay

Cell migration of SK-MEL-5 melanoma cells was investigated by making a cell-free gap with a 2 well culture- insert for 24 well plates (IbiTreat, Martinsried, Germany). The insert was made up of two wells that were separated by a thin wall. In each of the two wells of the insert, 70 μl of cell suspension containing 6×10^4^ cells was added. Cells were allowed to reach confluency for approximately 24 hours. The 2 well inserts were then removed and any resulting cell debris was washed with PBS. Fresh culture media containing either the vehicle control or deacetylnemorone was added to the cells, and the plates were incubated at 37 °C and 5% CO_2_ for 24 hours. Images were taken at 6, 12, and 24 hours after the addition of the treated media using a phase contrast Nikon Eclipse Ti-E inverted microscope. Percent invasion was calculated by measuring the gap distance at each time point and using the formula

$$Invasion\;\%=\frac{W0-Wn}{Wo}\times 100$$

in which Wn is the width of the gap at the desired time point, and W0 is the initial width zero right after forming a cell-free gap. One representative well was stained and imaged after the final time point. The media was first replaced with 400 μL of Cell Stain Solution (Cell Biolabs, Inc) and the plates were incubated for 15 minutes at room temperature. Each well was washed with deionized water and allowed to air dry. Images were taken using a phase contrast inverted microscope (Invitrogen EVOS FL Auto Cell Imaging). The invasion percent of each treatment group was determined in triplicate.

### Tube formation assay

Growth factor reduced BD Matrigel (Corning) was stored at -20 °C. Before use, the Matrigel was thawed on ice at 4 °C overnight. Next, 50 μL of Matrigel was added to each well of an ice-cold 96-well plate and incubated at 37 °C and 5% CO_2_ for 30 minutes, allowing a gel to form. A suspension of 20,000 HUVEC cells in 100 μL of cell culture media treated with deacetylnemorone was added to each well. The vehicle control contained only HUVEC cells suspended growth media. The junctions, or tubes, between the endothelial cells were imaged using an Invitrogen EVOS FL Auto at 4x magnification and manually counted. This process was done in triplicate for each treatment group.

## Results

### Deacetylnemorone induces concentration dependent cell death in immortalized cancer cell lines alone and in combination with FdUrd

**Fig 2:**
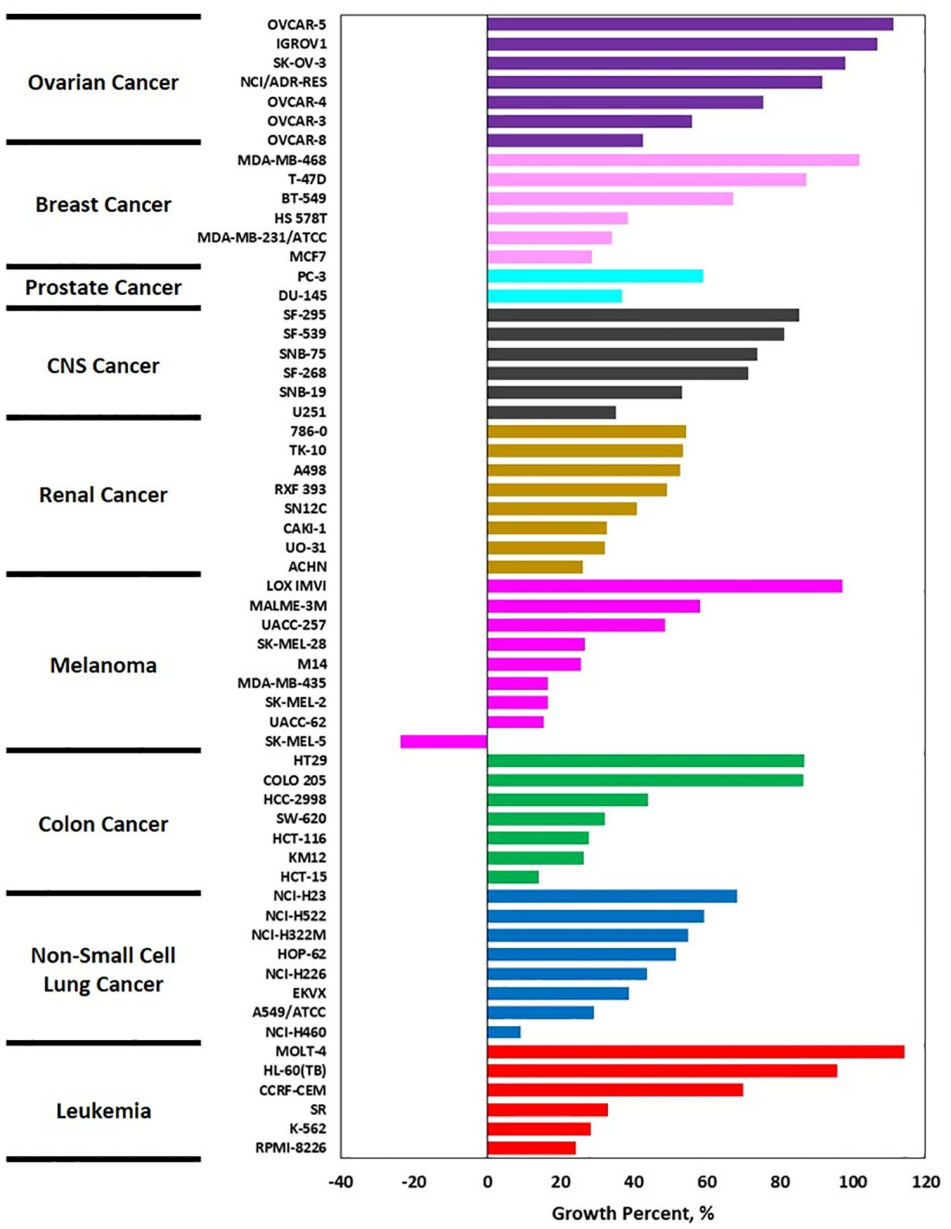
Waterfall plot of the growth percent of 59 cell lines in response to 10 μM of deacetylnemorone, determined by the NCI-60 one dose screening test. Growth percent was defined from -100%, where no cells were detected to 100%, where the cells were treated with growth media alone. Growth percent of 0% represented no change in the number of cells after treatment. The tissue type of each cell line is denoted by color (Purple–Ovarian Cancer, Pale pink–Breast Cancer, Aqua–Prostate Cancer, Grey–CNS cancer, Gold–Renal Cancer, Bright Pink–Melanoma, Green–Colon Cancer, Blue–Non-small cell lung cancer, Red–Leukemia).

In order to determine the chemotherapeutic potential of deacetylnemorone, the compound was screened against 59 cancer cell lines using the NCI-60 cancer panel. This panel utilizes a sulforhodamine B cell viability assay to determine the percent growth of cells treated with a compound of interest for 48 hours compared to cells treated with a vehicle control. Only one concentration, 10 μM, of deacetylnemorone was screened (Fig 2). This concentration inhibited the cell growth of 55 of the 59 cell lines tested, including at least one cell line from each of the 9 tissue types investigated. Of the 55 cell lines whose growth was inhibited by deacetylnemorone, one melanoma cell line, SK-MEL-5, exhibited cell death in response to 10 μM of the compound. The growth percent of SK-MEL-5 was -23.8% after 48 hours of treatment. While deacetylnemorone was capable of inhibiting the cell growth of each tissue type tested, it was particularly effective against melanoma, inhibiting the cell growth of four melanoma cell lines by at least 80%. This result suggested that the compound may be indicated for the treatment of melanoma.

**Fig 3:**
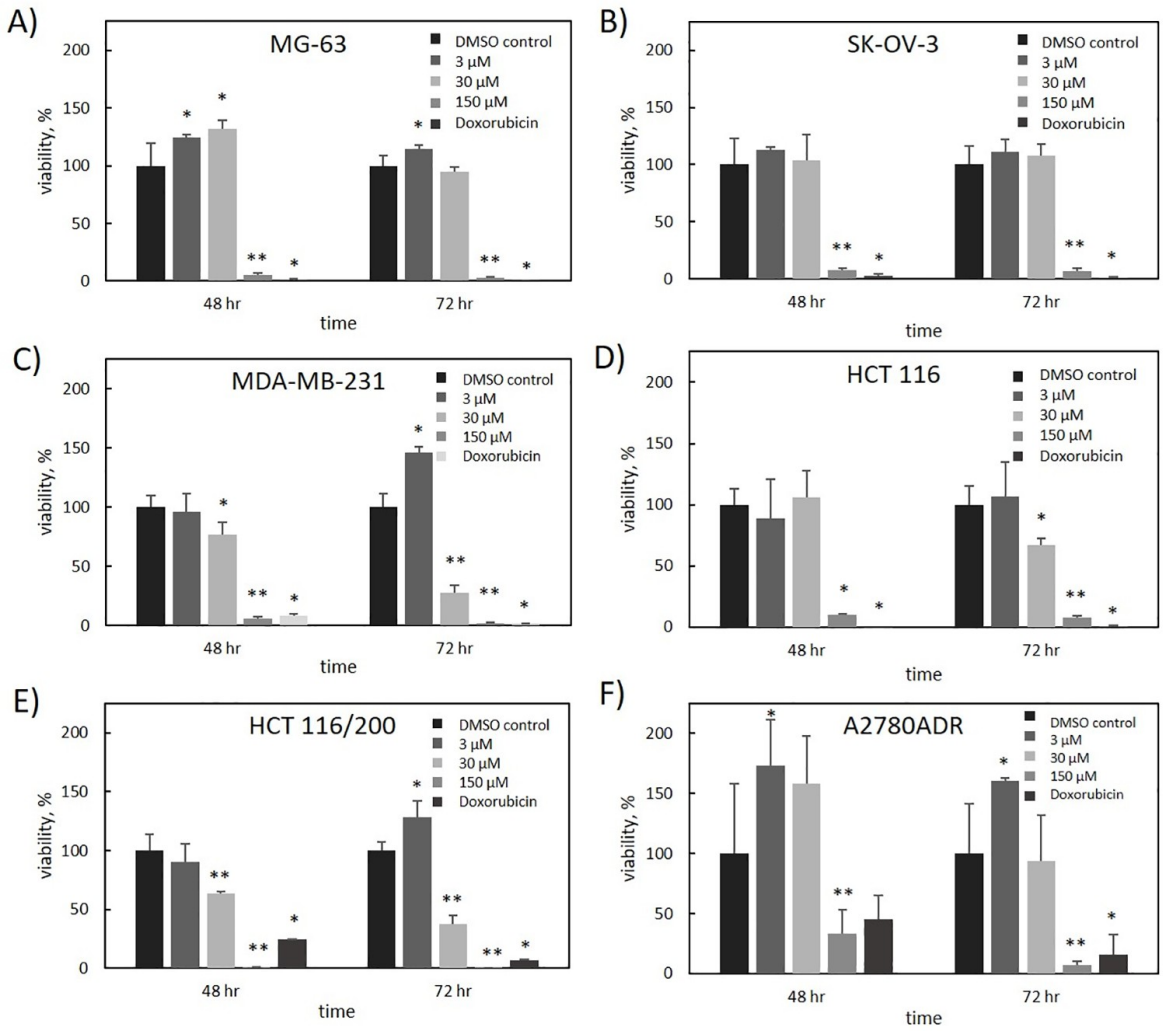
Percent viability of A) MG-63, B) SK-OV-3, C) MDA-MB-231, D) HCT 116, E) HCT 116/200, and F) A2780ADR cells in response to various concentrations of deacetylnemorone after 48 and 72 hours of exposure, as determined by the MTS cytotoxicity assay. For all cell lines except A2780ADR, the concentration of doxorubicin was 2 μM. For the A2780ADR cell line the concentration of doxorubicin was 1 μM. P-values, determined for each experimental group using a two-tailed t-test compared to the control, are shown above the bar graphs. * denotes a significant difference (p < 0.05) from the control group. ** denotes a significant difference (p < 0.05) from the previous concentration in addition to the control group. P-values greater than 0.05 were omitted.

**Fig 4:**
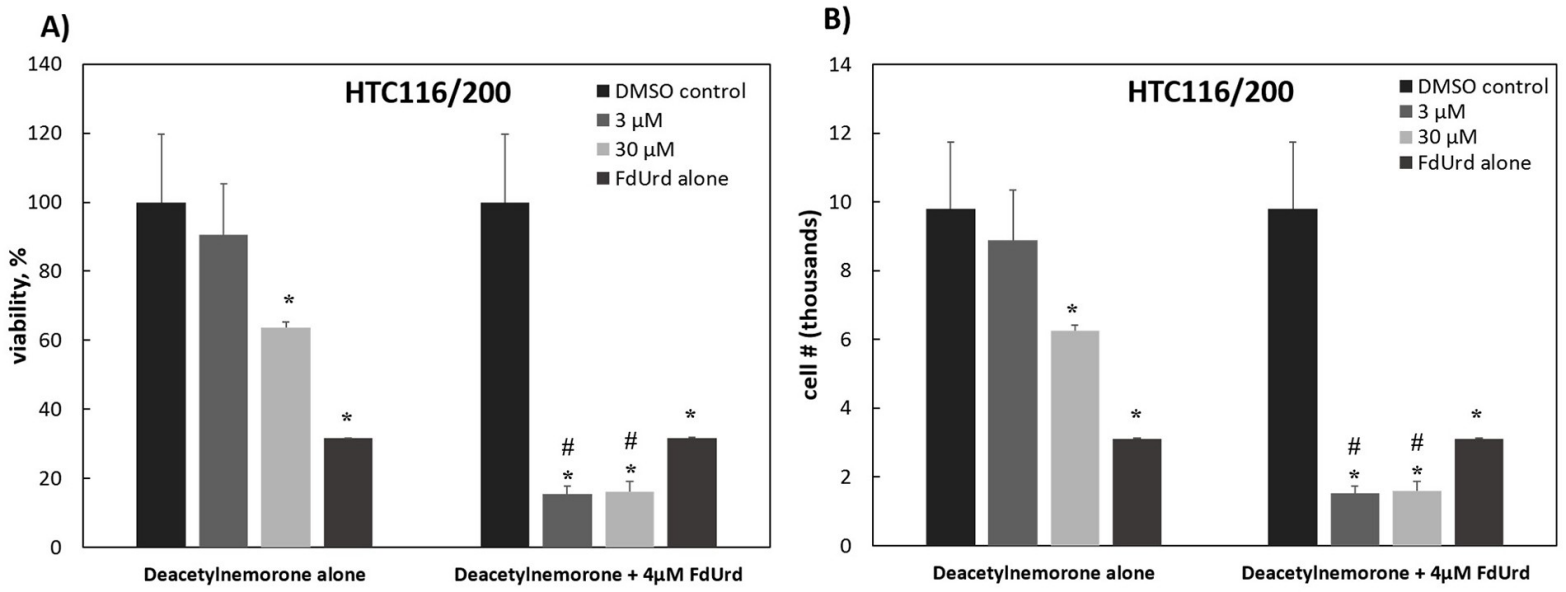
The A) percent viability and B) cell number of the HCT 116/200 cell line in response to a 48 hour exposure of various concentrations of deacetylnemorone, alone or in combination with the chemotherapy agent FdUrd, as calculated using the MTS assay. * denotes a viability significantly lower than the control. # denotes a viability significantly lower than the 4 μM FdUrd treatment. In all cases, significance is defined by a two tailed t-test with p < 0.05. P-values greater than 0.05 were omitted.

To further assess the growth inhibition properties of deacetylnemorone, a 3-dose MTS assay screen of 6 immortalized cancer cell lines was performed (Fig 3). The six cell lines examined were MG-63 (osteosarcoma), SK-OV-3 (ovarian cancer), MDA-MB-231 (breast cancer), HCT116 (colorectal carcinoma), HCT 116/200 (colorectal carcinoma), and A2780ADR (ovarian cancer). In each cell line tested, dose-dependent cell growth inhibition was observed; however, at low treatment concentrations, cell proliferation appeared to increase in some cell lines. This inhibition was significant in each cell line at concentrations less than or equal to 150 μM after 48 hours of treatment with deacetylnemorone. Notably, the inhibitory effect of the compound was stronger in HCT 116/200 cells than it was in HCT 116 cells. The HCT 116/200 cell line was derived from the HCT 116 cell line through treatment with gradually increasing concentration of the chemotherapeutic agent FdUrd[39]. This treatment induced resistance to FdUrd in the cell line compared to HCT116 by selecting for a resistant variant of thymidylate synthase. By comparing the doxorubicin control groups from Fig 3D and 3E, it was observed that the HCT116/200 cell line had also developed a cross-resistance to doxorubicin. The high sensitivity of the chemotherapy resistant HCT 116/200 cell line to deacetylnemorone when compared to the parent cell line prompted an investigation of the combinatorial effect of deacetylnemorone with FdUrd (Fig 4). Three concentrations of deacetylnemorone (3 μM, 30 μM, and 150 μM) were used to treat HCT 116/200 cells either alone or in combination with FdUrd. Each of these concentrations of deacetylnemorone significantly increased the growth inhibition of the 4μM FdUrd treatment. Both the deacetylnemorone-alone treatment and the combination treatment acted in a dose dependent manner.

### Deacetylnemorone delays progression of the cell cycle through S and G2/M phases

**Fig 5:**
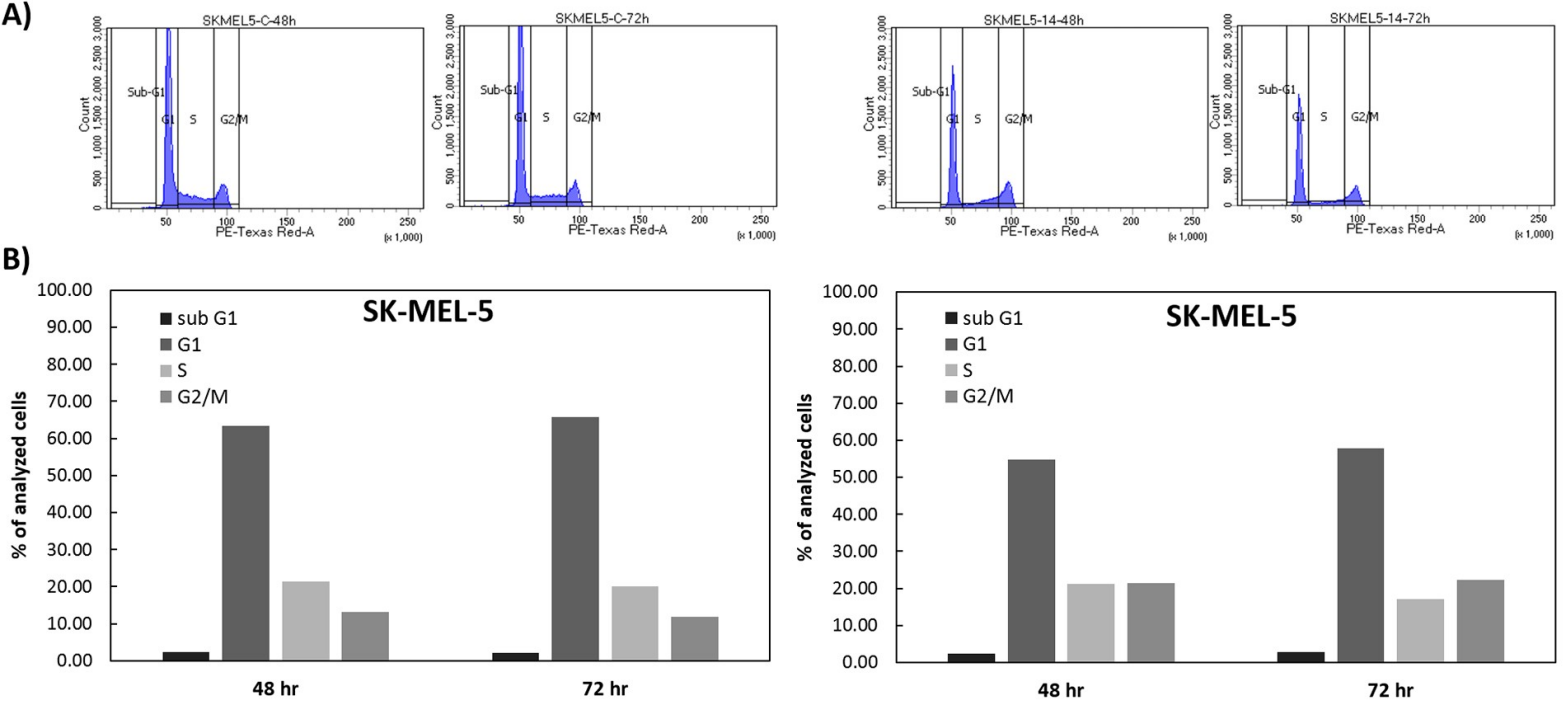
A) Histogram of propidium iodide expression as measured by flow cytometry for SK-MEL-5 cells treated with either a vehicle control or 15 μM of deacetylnemorone. The histograms were divided into four sections representing the sub-G1, G0/G1, S, and G2/M phases of the cell cycle. The histograms were used to calculate the percentage of analyzed cells treated with B) the vehicle control and C) 15 μM deacetylnemorone. This figure shows the cell cycle analysis only for 48 and 72H. The complete study for cell cycle analysis for all time points is in the supplementary material (S4 Fig).

Due to the growth inhibitory properties of deacetylnemorone, cell cycle analysis was performed on SKMEL5 melanoma cells exposed to the compound to gain insight into the mechanism of action. SKMEL5 cells were treated with deacetylnemorone for 72 hours, analyzing the DNA content of the cells at 6, 12, 24, 48, and 72 hours by propidium iodide (PI) staining followed by flow cytometry (Fig 5A). Using the generated histograms, the percentage of cells in the sub-G1, G0/G1, S, and G2/M phases of the cell cycle was determined (Fig 5B and 5C). Compared to the control, no increase in sub-G1 cells through the 72 hour treatment was observed, suggesting no apoptotic cell death was occurring. However, the treated group did exhibit a build-up of cells in the S-phase of the cell cycle through 24 hours of treatment, accompanied by a decrease in G0/G1 cells. This was followed by gradual decrease in S-phase cells and a subsequent increase in G2/M cells from 24 to 72 hours of treatment. These trends could be explained by a slowing down of progress through the cell cycle for the first 24 hours of treatment, followed by a gradual release of cells from the S-phase and G2-M phase between the 24 and 72 hour period. It is possible that higher concentrations of the compound could completely arrest progress through the cell cycle.

### Deacetylnemorone inhibits invasion of melanoma in vitro

**Fig 6:**
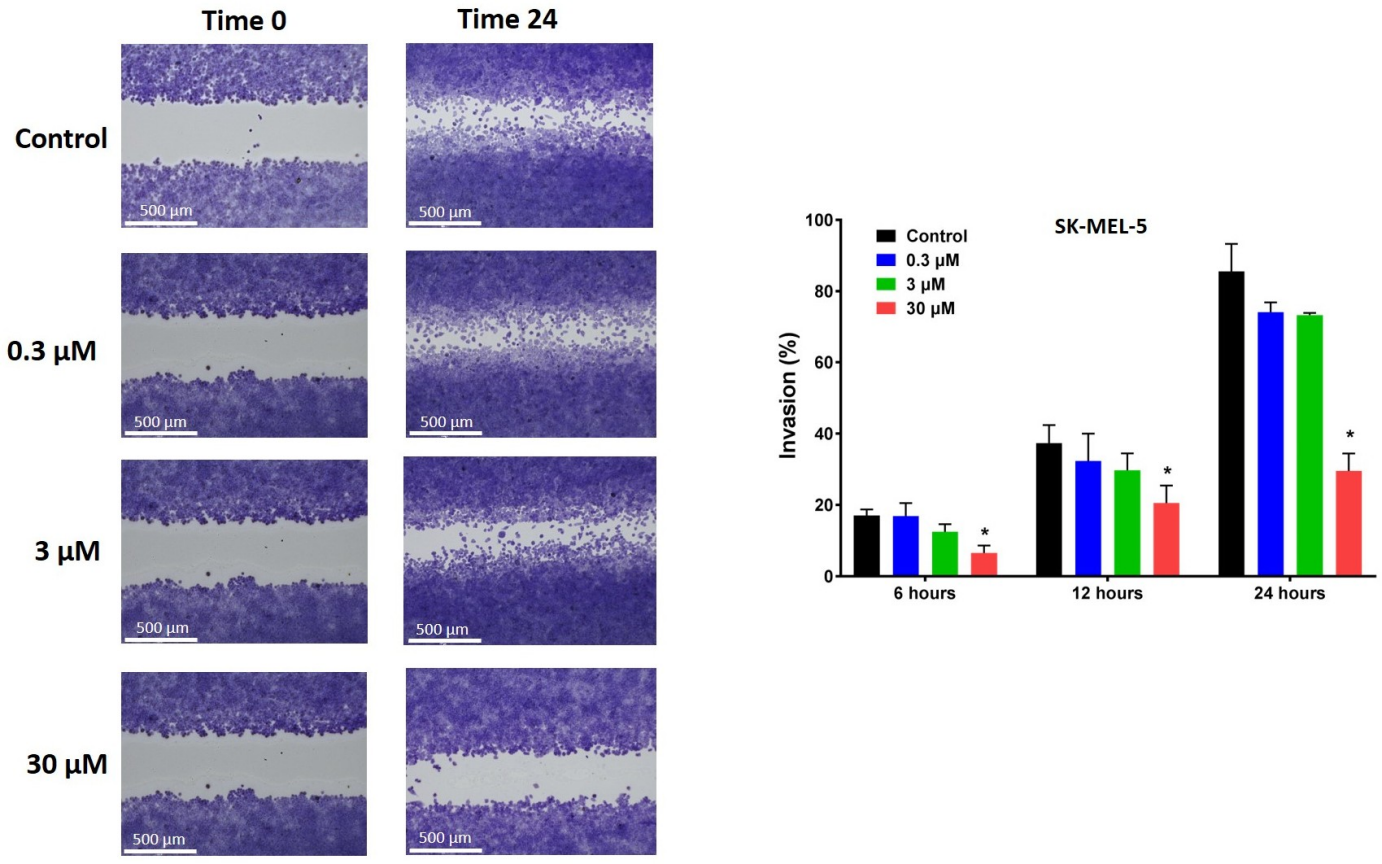
Invasion of SK-MEL-5 melanoma cells into a cell-free gap created using a 2-well cell culture insert when incubated with different concentrations of deacetylnemorone for 24 hours. A) A representative image of the cells at 0 and 24 hours after insert removal and the addition of deacetylnemorone. B) The percent invasion of the cells after 6, 12, or 24 hours. P values were determined using Ordinary-One-way ANOVA comparing each experimental group to the control group of the same time point. A significant difference, p ≤ 0.05, from the control at the same time point is denoted by *. P values greater than 0.05 were omitted from the fig.

The effect of deacetylnemorone on melanoma cell invasion was also investigated. A cell free gap was created between two regions of SK-MEL-5 melanoma cells using 2-well cell culture inserts. When the culture inserts were removed, the cells were treated for 24 hours with 0.3 μM, 3 μM, and 30 μM of deacetylnemorone. At 6, 12, and 24 hours the percent invasion into the cell free gap was measured (Fig 6). At each time point, the percent invasion of melanoma cells decreased as the concentration of deacetylnemorone increased. The inhibition of melanoma cell invasion was significantly lower (p < 0.05) than the control when the cells were treated with 30 μM deacetylnemorone at each of the tested time points (Fig 6B). Both the movement of the cell front and the migration of single cells into the cell free gap was inhibited as the concentration of deacetylnemorone was increased (Fig 6A). Trypan blue cytotoxicity assays were also performed at each time point, revealing the inhibition of cancer cell invasion occurred at lower concentrations of deacetylnemorone than was toxic to the cells (S3 Fig).

### Deacetylnemorone inhibits tube formation of endothelial cells, a critical step of angiogenesis

**Fig 7:**
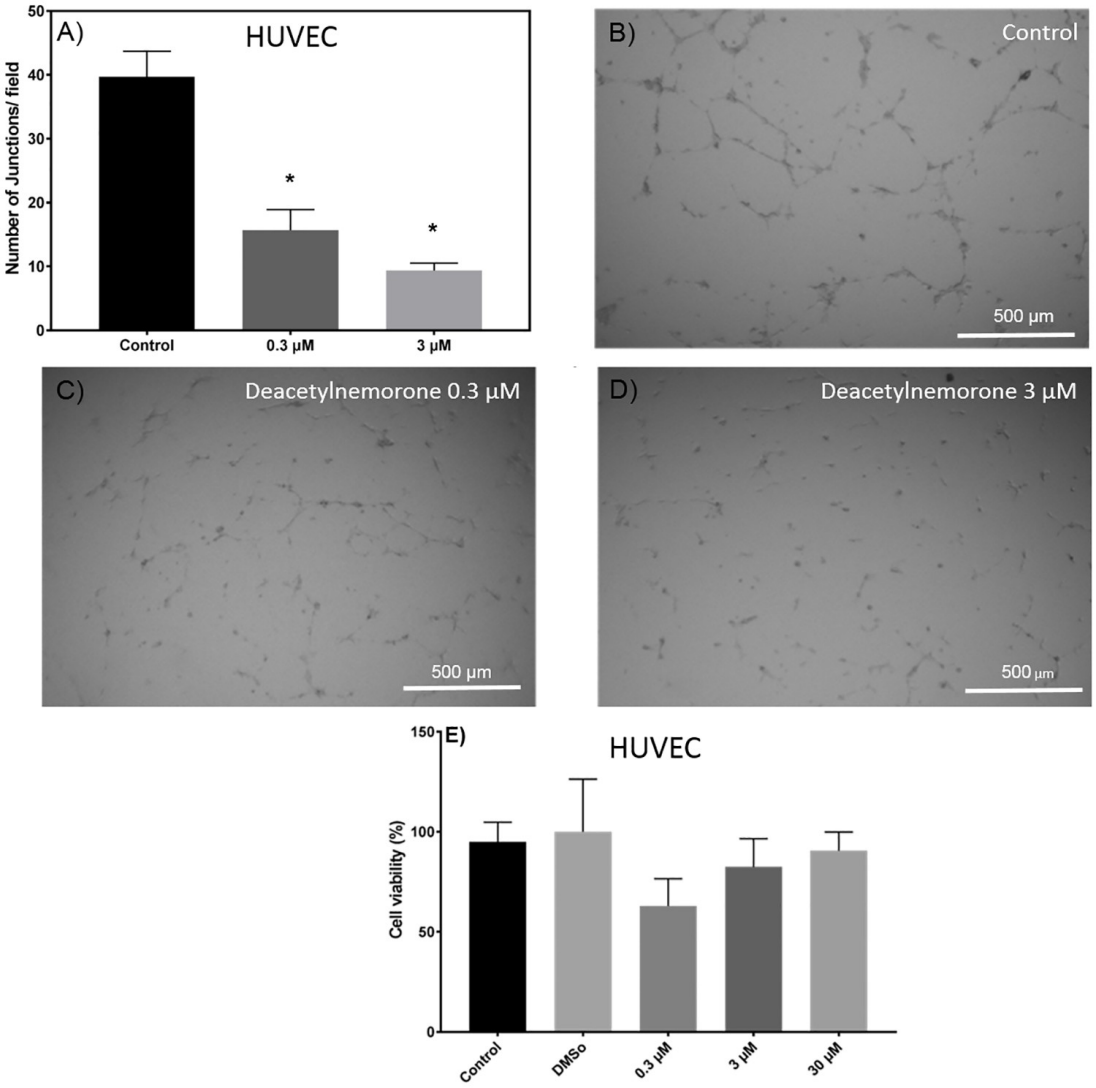
A) The average number of junctions, or tubes, formed between HUVEC endothelial cells after 8 hours of incubation on growth factor reduced BD Matrigel. A representative bright field image is shown for treatment with B) growth media alone, C) 0.3 μM deacetylnemorone, and D) 3 μM deacetylnemorone. E) The percent viability of HUVEC endothelial cells in response to 8 hours of incubation with various concentrations of deacetylnemorone as determined by an MTS assay. The control group was treated with culture media alone, and the DMSO group was treated with 0.05% DMSO in culture media. P values were determined using multiple t-tests comparing each treated group to the control. * represents a significant difference (p < 0.05) from the control group. P values greater than 0.05 were omitted from the fig.

The effect of deacetylnemorone on angiogenesis was investigated using a tube formation assay (Fig 7). The assay consisted of HUVEC endothelial cells grown on a Matrigel basement membrane in the presence of growth factors. Under these conditions, cellular projections called “tubes” will begin to form between the cells (Fig 7B). In this study, tube formation was allowed to occur for 8 hours under control conditions or with growth media treated with deacetylnemorone. The number of tubes or junctions per field were manually counted in triplicate for the groups treated with the control (culture media alone), 0.3 μM deacetylnemorone, and 3 μM deacetylnemorone, and a dose-dependent decrease in tube formation between HUVEC endothelial cells was observed as the concentration of deacetylnemorone was increased (Fig 7A). The decrease for both the group treated with 0.3 μM deacetylnemorone and the group treated with 3 μM deacetylnemorone was significant (p ≤ 0.05) when compared to the control treated group. A representative image of the cells treated with 0.3 μM and 3 μM deacetylnemorone can be seen in Fig 7C and 7D respectively. The toxicity of an 8 hour treatment of deacetylnemorone on HUVEC endothelial cells was also determined using the MTS assay. No significant difference (p ≤ 0.05) in cell viability between HUVEC cells treated with the vehicle control and the HUVEC cells treated with up to 30 μM deacetylnemorone was observed (Fig 7E), indicating the compound was not cytotoxic to HUVEC cells at the concentrations used to inhibit angiogenesis.

## Discussion

Multi-targeting natural products may create renewed vigor in the use of natural compounds for the treatment of cancer. Targeted therapies hold great promise for the future of cancer treatment but have been accompanied by numerous shortcomings, including high rates of resistance, low rates of susceptible patients, and high cost. Natural products that attack multiple cancer-related pathways may limit therapy-induced resistance and provide robust treatment when in combination with currently available therapies. Deacetylnemorone was examined in this study to determine its ability to interfere with multiple cancer-related pathways, including cancer cell growth and proliferation, cancer cell invasion, and angiogenesis.

The growth inhibitory properties of deacetylnemorone were first examined by submitting the compound to the NCI-60 one dose cytotoxicity screen. At 10 μM, deacetylnemorone exhibited growth inhibitory properties across all nine of the tissue types examined. Within each tissue type a range of activity was observed, from no growth inhibition at all to inducing cell death in one melanoma cell line. These results suggest that the growth inhibitory effects of deacetylnemorone are not tissue-type dependent. However, the compound did appear to have a strong effect on multiple cell lines isolated from melanoma. SK-MEL-5 is a melanoma cell line that exhibited a 23.8% reduction in cell viability after 48 hours of treatment with deacetylnemorone. As a result, it appears that at concentrations near 10 μM, deacetylnemorone is selective in inducing cell death. The growth inhibition and cytotoxicity results across the 59 cell lines tested in the NCI-60 screen were less potent than other compounds that have undergone NCI-60 screening, and thus further screening was not performed using the NCI-60 panel. However, dose-dependent growth inhibition was demonstrated independently using an MTS viability assay on MG-63, SK-OV-3, A2780ADR, MDA-MB-231, HCT 116, and HCT 116/200 cells. Each of the six cell lines tested exhibited a dose-dependent response in cell viability to deacetylnemorone. Some cell lines appeared to exhibit increased proliferation in response to low concentrations of deacetylnemorone. As the viability was determined using an MTS assay based upon metabolic activity this apparent overestimation of viability could be explained by a survival response of the cells increasing metabolic activity, reduction of the tetrazolium salt by deacetylnemorone, or the increased number of mitochondria per cell present as a result of the observed slowdown of cell cycle progression through S and G2/M phase [40]. Despite this, in each case, the viability of the cells treated with 150 μM of deacetylnemorone was significantly less than the control (p ≤ 0.05). In the case of colorectal carcinoma (HCT 116 and HCT 116/200) and breast cancer (MDA-MB-231), a significant decrease (p ≤ 0.05) compared to the control group at 30 μM of deacetylnemorone was observed. This confirmed the selectivity noted in the NCI-60 screening.

Of particular interest was the seemingly greater sensitivity of the chemotherapy resistant HCT116/200 cells to deacetylnemorone compared with the parent HCT 116 cell line. Deacetylnemorone at 30 μM reduced the cell viability of HCT 116/200 after only 48 hours compared to the 72 hours needed for the parent cell line. The compound also reduced the cell viability of the HCT 116/200 cell line to a greater extent than the parent cell line at both the 30 and 150 μM concentrations. The compound may therefore be targeting a cellular pathway that is responsible for the treatment-induced resistance of HCT 116/200 cells or was co-selected with the cellular pathway responsible for the treatment-induced resistance such as the percentage of cancer stem cells within the population[41]. In order to further explore this interesting result, deacetylnemorone was used in combination with the chemotherapy agent FdUrd to treat HCT 116/200 cells. The cell viability of HCT 116/200 was significantly lower when deacetylnemorone was used in combination with FdUrd than when FdUrd was used alone. This effect occurred at as little as 3 μM deacetylnemorone, a concentration lower than the minimum required to reduce cell viability when deacetylnemorone was used on this cell line alone. It is likely that a synergistic rather than simply a combinatorial effect occurred when deacetylnemorone was used alongside the chemotherapy.

Next, cell cycle analysis was performed to gain insight into the mechanism of action for the cell growth inhibition of SK-MEL-5 melanoma cells. When compared to the control, deacetylnemorone at 15 μM did not increase the percentage of cells in the sub G1 phase of the cell cycle, suggesting apoptotic cell death was likely not occurring. As a result, another mechanism of cell death, such as necrosis or autophagy, is likely responsible for any cell death observed at this dose[42]. Additionally, from 6 to 24 hours of incubation with deacetylnemorone, the percentage of cells in the G1 phase decreased with a corresponding increase in the S phase cells. After 24 hours, the percent of S-phase cells decreased and the percentage of cells in the G2/M phase increased. The increase in G2/M phase cells continued until 72 hours of incubation with deacetylnemorone, at which point the percentage of cells in the G2/M phase outnumbered the percentage of cells in the S-phase. This trend was not seen on cells treated with the vehicle control. These results suggest that deacetylnemorone may slow progression of the cell cycle through the S and G2/M phases. While the mechanism is unclear, this could be the result of DNA damage, inhibition of DNA synthesis, or inhibition of cell cycle regulating cyclins[43].

With the cancer cell growth inhibition properties of deacetylnemorone established, the effect of the compound on other cancer-related pathways was then examined. Deacetylnemorone was observed to reduce SK-MEL-5 invasion at a concentration as low as 0.3 μM after 24 hours of treatment. The decrease in invasion was concentration dependent, and the decrease became significant (p ≤ 0.05) at 30 μM of the compound. Both the migration of the cell front and the invasion of single cells into a cell free gap between cell fronts were inhibited as deacetylnemorone was added to the cell culture media. The inhibition of cell front migration could be interpreted as an extension of the cell growth inhibition observed previously, as the cell front will migrate when the cells divide. However, the decrease in single cells invading the cell free gap suggests that the cells were being inhibited from undergoing epithelial-mesenchymal transition (EMT). This process allows cancer cells to detach from their extracellular matrix, move freely within the body, and reattach in a new location, establishing metastatic growth[44]. Cancer cells undergoing this process may also be linked to innate chemotherapy resistance and an increased percentage of cancer stem cells[41]. The decrease of single cells in the cell free gap suggests a decrease in the number of cells that had migrated from one of the cell fronts. This inhibition is unique from cell growth inhibition and may lead to an ability of deacetylnemorone to inhibit the metastasis and chemotherapy resistance of melanoma. The final cancer-related pathway that was assayed for a response to deacetylnemorone was angiogenesis. At sub-cytotoxic concentrations of deacetylnemorone (0.3 and 3 μM), significantly less tube formation was observed between HUVEC endothelial cells. Tube formation is a crucial step in angiogenesis, which is required for both extended tumor growth and metastatic formation. By inhibiting the formation of tubes between endothelial cells, deacetylnemorone may cut off the blood supply to new and growing tumors and further inhibit metastasis.

## Conclusions

Deacetylnemorone is a natural product of the abietane diterpenoid family. While limited growth inhibition studies have been performed to investigate the potential of this compound, it has remained an understudied lead compound for anti-cancer therapy. In this study, deacetylnemorone was shown to inhibit cell growth of a wide variety of cancer cell lines, induce non-apoptotic cell death in SK-MEL-5 melanoma, sensitize HCT 116/200 resistant colorectal carcinoma to chemotherapeutic treatment, inhibit the EMT and invasion of melanoma cells, and inhibit angiogenesis. While no specific cellular targets of deacetylnemorone were identified in this study, the range of diverse biological effects of the compound suggest that it is interfering with multiple cancer related pathways. As a result, it is likely that deacetylnemorone interacts with multiple cellular targets rather than one specific protein. Future studies should seek to elucidate the exact cellular mechanisms of the anti-cancer effects demonstrated by this study. These properties may give deacetylnemorone the ability to provide a robust, multi-targeted treatment for a range of cancers, which not only increases the efficacy of current cancer treatment combinations and reduces the risk of treatment-acquired resistance, but also re-sensitizes already resistant tumors to further chemotherapy use. Further examination is warranted to elucidate the mechanism by which each of deacetylnemorone’s anti-cancer effects are produced in addition to translating these *in vitro* results *in vivo*. Additionally, the ability of deacetylnemorone to target cancer stem cells specifically should be investigated as a potential mechanism for the ability of the compound to inhibit cell growth in chemotherapy resistant cell lines and inhibit EMT. In summary, deacetylnemorone is a multi-targeted natural product which has the potential to enhance currently utilized cancer treatments when used in combination with chemotherapeutic, anti-angiogenic, and other targeted therapies.

## Supporting information

S1 Fig The A) ^1^H-NMR, B) ^13^C-NMR, C) H-H COSY, D) HSQC, and E) HMBC spectra of the abietane diterpenoid, deacetylnemorone (in DMSO-d6). (DOCX)

S2 Fig The A) negative ion mode time of flight-mass spectrometry and B) negative mode HR-MS spectra of deacetylnemorone used to determine the mass. (DOCX)

S3 Fig Viability of SK-MEL-5 cells after 6, 12, or 24 hours of incubation with deacetylnemorone. The cells were seeded in 2 well culture inserts within 24 well culture plates and allowed to reach confluency before being treated with deacetylnemorone. Viability was determine by manually counting cells excluding trypan blue using a hemocytometer. (Note no data was collected for the 30μM concentration at 24 hours).
(DOCX)

S4 Fig SK-MEL-5 cell cycle analysis after deacetylnemorone treatment at 6, 12, 24, 48, and 72 H. A) Histogram of propidium iodide expression as measured by flow cytometry for SK-MEL-5 cells treated with either a vehicle control or 15 μM of deacetylnemorone. The histograms were divided into four sections representing the sub-G1, G0/G1, S, and G2/M phases of the cell cycle. The histograms were used to calculate the percentage of analyzed cells treated with B) the vehicle control and C) 15 μM deacetylnemorone.
(DOCX)

S1 Table 1H and 13C NMR data (400 and 100 MHz, in DMSO-d6) of compound deacetylnemorone. (DOCX)
