# Supplementary material for: A multi-targeting natural compound with growth inhibitory and anti-angiogenic properties re-sensitizes chemotherapy resistant cancer
Source: PLoS One. 2019 Jun 11;14(6):e0218125. doi: 10.1371/journal.pone.0218125 (PMC6559640; doi:10.1371/journal.pone.0218125)
Supplement: S2 Fig — (DOCX) [file pone.0218125.s002.docx]

**A multi-targeting natural compound with growth inhibitory and anti-angiogenic properties re-sensitizes chemotherapy resistant cancer**

**Supplementary Figures**

**A)**

**
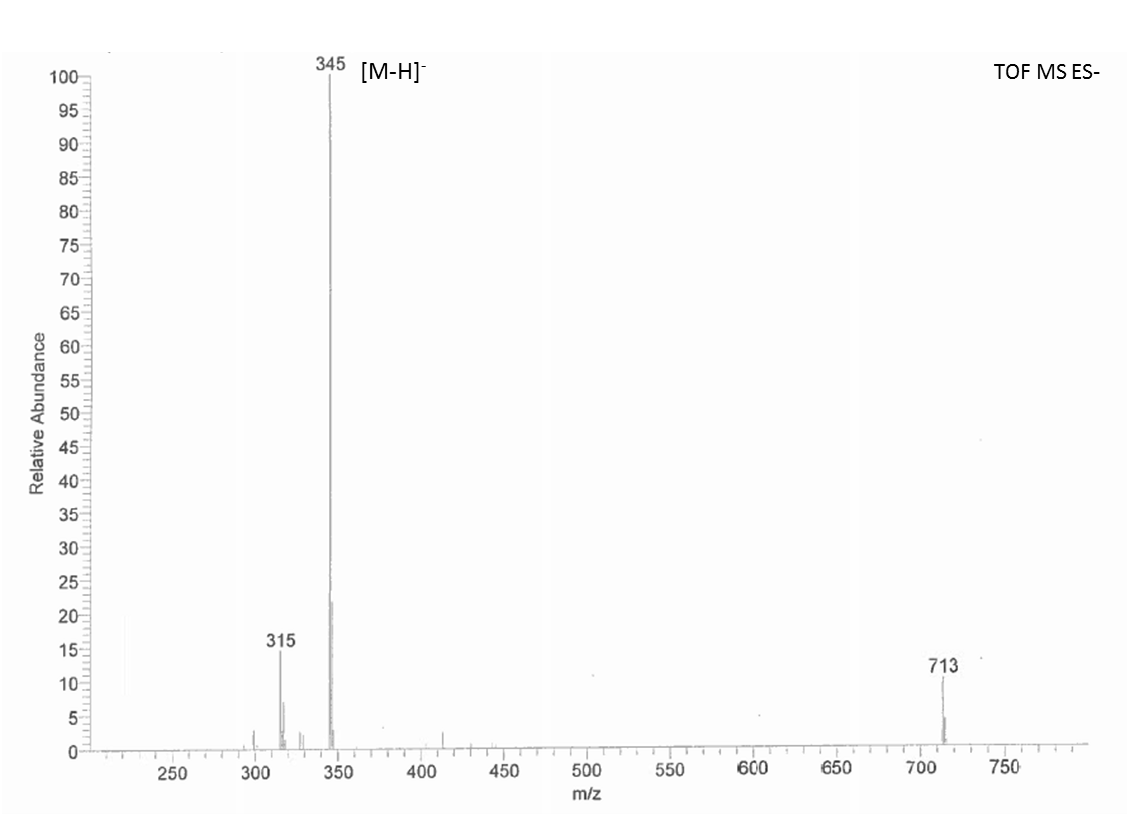
**

**B)**

**
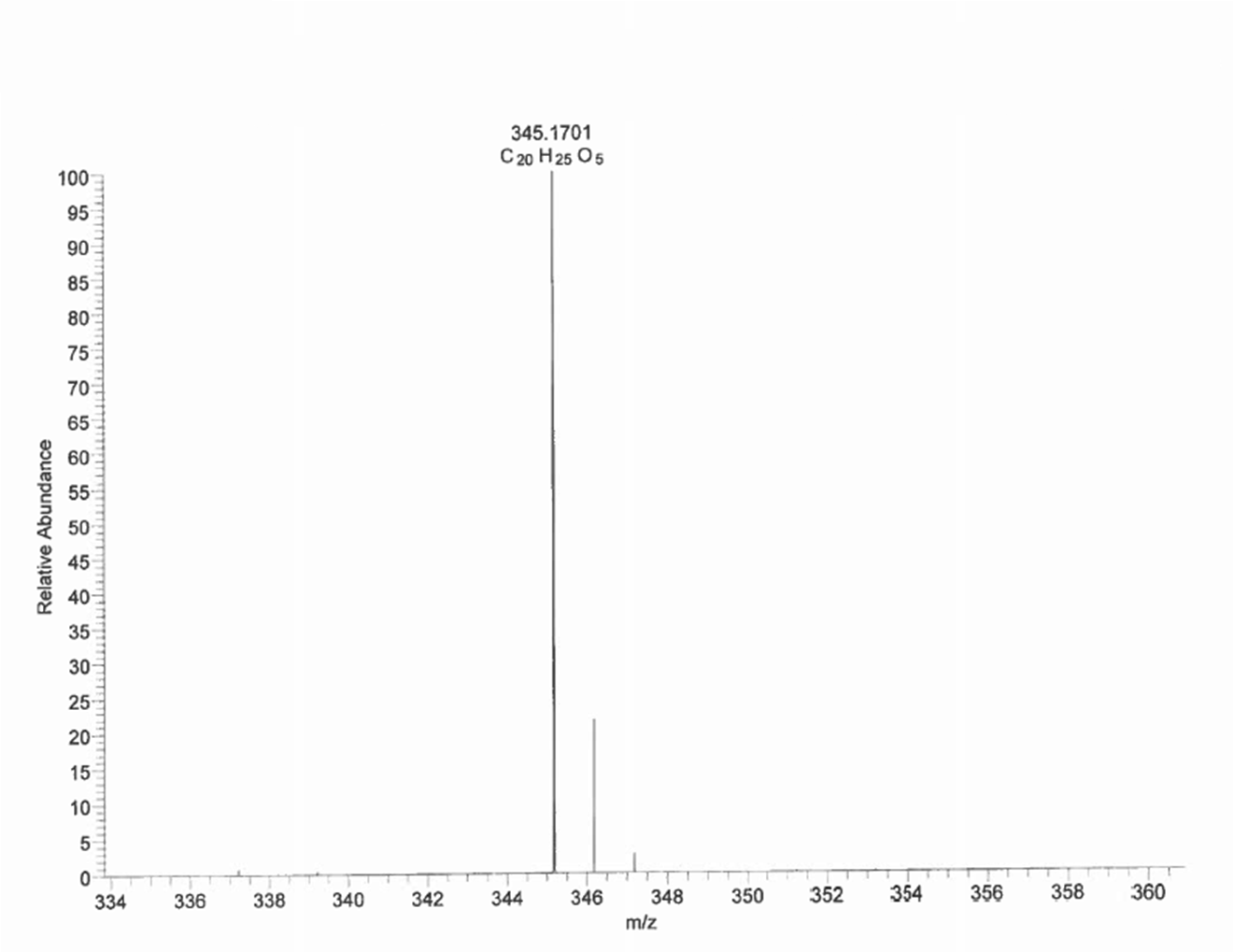
**

**S2 Fig.**
