# Supplementary material for: A multi-targeting natural compound with growth inhibitory and anti-angiogenic properties re-sensitizes chemotherapy resistant cancer
Source: PLoS One. 2019 Jun 11;14(6):e0218125. doi: 10.1371/journal.pone.0218125 (PMC6559640; doi:10.1371/journal.pone.0218125)
Supplement: S3 Fig — The cells were seeded in 2 well culture inserts within 24 well culture plates and allowed to reach confluency before being treated with deacetylnemorone. Viability was determine by manually counting cells excluding trypan blue using a hemocytometer. (Note no data was collected for the 30μM concentration at 24 hours). (DOCX) [file pone.0218125.s003.docx]

**A multi-targeting natural compound with growth inhibitory and anti-angiogenic properties re-sensitizes chemotherapy resistant cancer**

**Supplementary Figures**


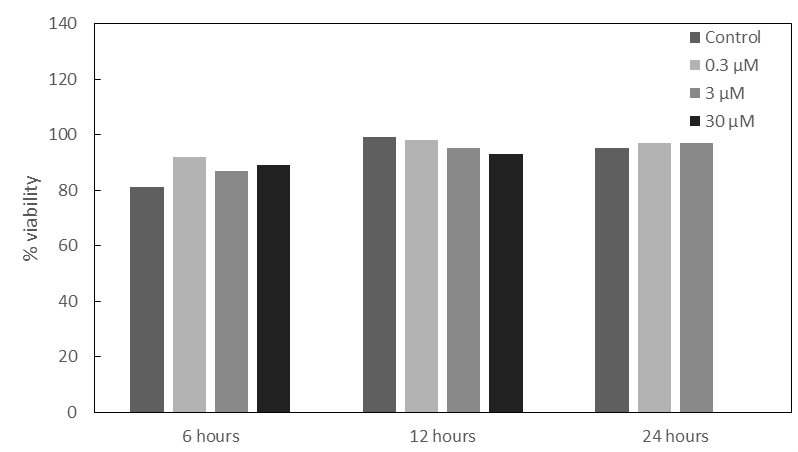


**S3 Fig.**
