# Supplementary material for: A multi-targeting natural compound with growth inhibitory and anti-angiogenic properties re-sensitizes chemotherapy resistant cancer
Source: PLoS One. 2019 Jun 11;14(6):e0218125. doi: 10.1371/journal.pone.0218125 (PMC6559640; doi:10.1371/journal.pone.0218125)
Supplement: S4 Fig — A) Histogram of propidium iodide expression as measured by flow cytometry for SK-MEL-5 cells treated with either a vehicle control or 15 μM of deacetylnemorone. The histograms were divided into four sections representing the sub-G1, G0/G1, S, and G2/M phases of the cell cycle. The histograms were used to calculate the percentage of analyzed cells treated with B) the vehicle control and C) 15 μM deacetylnemorone. (DOCX) [file pone.0218125.s004.docx]

**A multi-targeting natural compound with growth inhibitory and anti-angiogenic properties re-sensitizes chemotherapy resistant cancer**

**Supplementary Figures**


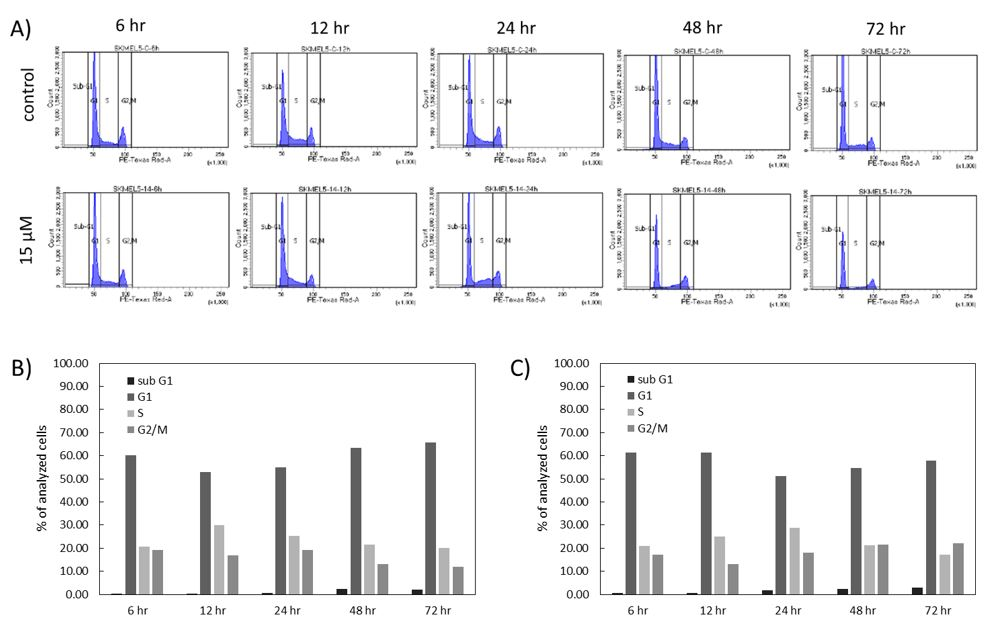


**S4 Fig.**
