# Supplementary material for: A multi-targeting natural compound with growth inhibitory and anti-angiogenic properties re-sensitizes chemotherapy resistant cancer
Source: PLoS One. 2019 Jun 11;14(6):e0218125. doi: 10.1371/journal.pone.0218125 (PMC6559640; doi:10.1371/journal.pone.0218125)
Supplement: S1 Table — (DOCX) [file pone.0218125.s005.docx]

**A multi-targeting natural compound with growth inhibitory and anti-angiogenic properties re-sensitizes chemotherapy resistant cancer**

**Supplementary Figures**

**S1 Table.**

| **position** | **δ_H_** | **δ_C_** |
| --- | --- | --- |
| 1^a^ | 1.26 (dd) | 31.6 |
| 1^b^ | 2.76 (dd) | 31.6 |
| 2^a^ | 1.63 (m) | 19.01 |
| 2^b^ | 2.03 (m) | 19.01 |
| 3^a^ | 1.31 (dd) | 40.7 |
| 3^b^ | 1.51 (dd) | 40.7 |
| 4 | - | 33.2 |
| 5 | 1.97 (dd) | 47.3 |
| 6a | 1.66 (m) | 26.4 |
| 6b | 1.66 (m) | 26.4 |
| 7 | 4.70 (t) | 59.9 |
| 8 | - | 144.7 |
| 9 | - | 143.03 |
| 10 | - | 57.6 |
| 11 | - | 183.8 |
| 12 | - | 153.2 |
| 13 | - | 125.2 |
| 14  18 | - | 186.3 |
| 15 | 3.11 (sep) | 24.0 |
| 16 | 1.13 (d) | 20.1 |
| 17 | 1.17 (d) | 20.4 |
| 18 | 0.67 (s) | 22.9 |
| 19 | 0.91 (s) | 31.4 |
| 20 | 10.05 (s) | 204.1 |
